# Treatment-Free Remission in Chronic Idiopathic Thrombocytopenic Purpura

**DOI:** 10.7759/cureus.8705

**Published:** 2020-06-19

**Authors:** Mohanad Ahmed, Mohamed A Yassin

**Affiliations:** 1 Internal Medicine, Hamad Medical Corporation, Doha, QAT; 2 Hematology and Oncology, Hamad General Hospital, Doha, QAT

**Keywords:** eltrombopag, itp in pediatric, treatment-free remission

## Abstract

Idiopathic thrombocytopenic purpura (ITP) is a disease in which the immune system attacks platelets and causes a decrease in their number, exposing the patient to bleeding risk. It is a diagnosis by exclusion. ITP usually presents as acute disease and is self-limiting in pediatric patients, while it tends to be chronic in adults. Eltrombopag is a thrombopoietin receptor agonist used as a second-line treatment for ITP. This drug is approved for use in adults as second-line therapy, but little is known about its use in the pediatric patient population. We report the case of a 14-year-old girl with chronic steroid-dependent ITP who responded well to eltrombopag and maintained treatment-free remission after stopping the drug.

## Introduction

Idiopathic thrombocytopenic purpura (ITP) in childhood is characterized by isolated thrombocytopenia (i.e., platelet count <100,000/microliter with normal white blood cell count and hemoglobin) [1]. Initial management in children with newly diagnosed ITP may consist of either "watchful waiting" or pharmacologic intervention. First-line treatment options include glucocorticoids, intravenous immune globulin, and intravenous anti-D immune globulin. Most children with ITP recover within three to six months from the time of presentation, with or without treatment. Approximately 10% to 20% of affected children develop chronic ITP, defined as thrombocytopenia for more than 12 months from presentation. For patients with chronic ITP whose symptoms are not adequately controlled using first-line therapies and for those who remain dependent on glucocorticoids for symptom control, treatment with either rituximab or a thrombopoietin receptor (TPO-R) agonist (e.g., eltrombopag, romiplostim) is suggested [1].

Approximately 10 years after its initial approval for adult chronic ITP, eltrombopag was approved to treat pediatric patients with chronic ITP [2,3]. Long-term safety data on the use of eltrombopag in children are limited, and studies in adults have not revealed a clinically significant increase in the incidence of thrombosis [3]. We report our experience treating a child with steroid-dependent ITP, who maintained treatment-free remission after stopping the drug without developing significant side effects like nausea or vomiting.

## Case presentation

**Figure 1:**
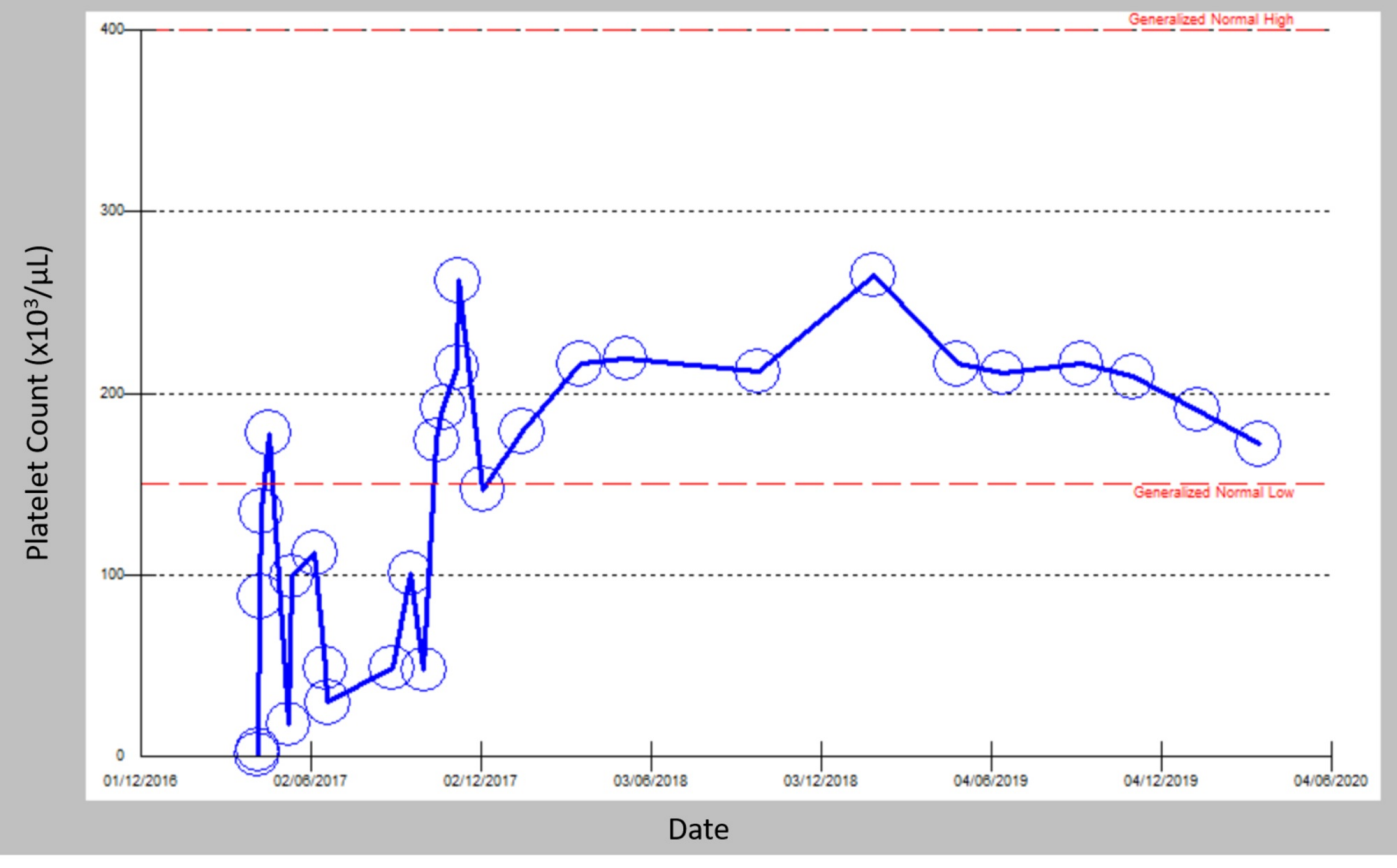
Change in platelet count from 2017 to 2020

A 14-year-old girl presented with bleeding gums in April 2017. She had no history of chronic illness. She was found to have a platelet count of 1 and was diagnosed with ITP by exclusion. She was admitted to the hospital and started on intravenous steroids for three days. Her platelet count improved to 135, and she was subsequently discharged. One month later, she presented again with bleeding gums and a platelet count of 18; a diagnosis of chronic ITP was reached by exclusion, and she was given intravenous steroids with improvement in her platelet count to 112. She was discharged on oral steroids at a dose of 1 mg/kg. Her platelet count was acceptable in follow-up outpatient visits. After two months of tapering and eventually stopping steroids, her platelet count dropped to 18. At that point, a discussion about second-line therapy was raised, and options were discussed with the patient and her family, who opted for eltrombopag. In October 2017, the patient was started on eltrombopag 50 mg daily and closely followed over the next few weeks in the outpatient clinic. The patient maintained adequate platelet counts with no side effects. Two months later, the dose was decreased to 25 mg daily and the patient continued to have adequate platelet counts with no side effects. After two years of using the drug (late 2019), eltrombopag was stopped, and her platelet count was monitored over the following eight months from September 2019 till last check in April 2020 (Figure 1). The patient showed treatment-free remission for the last eight months.

## Discussion

This patient falls within the 20% category of patients with ITP who develop the chronic form of the disease. She responded well to steroids but eventually became steroid-dependent. Steroids have a serious side effect profile; they can cause Cushing's syndrome, reactivation of infections, and mineral bone disease, in addition to other metabolic side effects [4]. To avoid exposing our patients to such adverse events, a discussion was held with the patient and her family regarding starting eltrombopag for the purpose of controlling her symptoms and platelet count.

Eltrombopag is small-molecule nonpeptide TPO-R agonist administered orally. It can effectively increase platelet counts and reduce bleeding events in patients with chronic ITP, with an overall response rate of 60% to 80% [5]. Eltrombopag is well tolerated and has a good safety profile in adults [5]. It is recommended for splenectomized patients with ITP who are refractory to other treatments (e.g., corticosteroids, immunoglobulins). It may also be considered as a second-line treatment modality for adult nonsplenectomized patients who refuse surgery or for whom surgery is contraindicated [5].

Upon review of the literature, we found that eltrombopag is not recommended for children and adolescents younger than 18 years due to insufficient data on its safety and efficacy [5]. Nonetheless, our patient was started on eltrombopag, and she maintained adequate platelet counts and remained asymptomatic throughout the treatment period. Even after stopping the drug, she had treatment-free remission, and her symptoms and platelet count remained stable.

## Conclusions

Eltrombopag can be used safely in children with chronic ITP and can help achieve treatment-free remission. However, further studies are needed to reproduce this finding.
